# Dual-Stream Fusion Network with ConvNeXtV2 for Pig Weight Estimation Using RGB-D Data in Aisles

**DOI:** 10.3390/ani13243755

**Published:** 2023-12-05

**Authors:** Zujie Tan, Junbin Liu, Deqin Xiao, Youfu Liu, Yigui Huang

**Affiliations:** 1 College of Mathematics Informatics, South China Agricultural University, Guangzhou 510642, China; tanzujie1997@stu.scau.edu.cn (Z.T.); liu@stu.scau.edu.cn (J.L.); lyf0313@126.com (Y.L.); hyg2021scau@stu.scau.edu.cn (Y.H.); 2 Key Laboratory of Smart Agricultural Technology in Tropical South China, Ministry of Agriculture and Rural Affairs, Beijing 100125, China

**Keywords:** computer vision, deep learning, mass measurement

## Abstract

**Simple Summary:**

In the realm of livestock management, accurately estimating the weight of pigs presents a critical yet challenging task, particularly in the dynamic environment of farms. Traditional methods often struggle due to the continuous movement of pigs and fluctuating conditions such as lighting. To address these challenges, our study focuses on developing a novel method that simplifies weight estimation while adapting to the constantly changing conditions of real-world pig farms. Our solution, the moving pig weight estimate algorithm based on deep vision (MPWEADV), marks a significant step in this direction. It employs advanced imaging technology to capture both the visual appearance and depth information of moving pigs. The central idea is to combine these two types of data for more accurate weight estimates than traditional methods could provide. To validate our proposed method, we replicated two recently published methods and demonstrated through experimental results that our pig weight estimation model could rapidly and accurately determine the weight of pigs in the challenging scenarios we constructed. This model operates in an unconstrained environment, providing real-time evaluation of pigs’ weight, thereby offering data support for grading and adjusting breeding plans, indicating a wide range of potential applications.

**Abstract:**

In the field of livestock management, noncontact pig weight estimation has advanced considerably with the integration of computer vision and sensor technologies. However, real-world agricultural settings present substantial challenges for these estimation techniques, including the impacts of variable lighting and the complexities of measuring pigs in constant motion. To address these issues, we have developed an innovative algorithm, the moving pig weight estimate algorithm based on deep vision (MPWEADV). This algorithm effectively utilizes RGB and depth images to accurately estimate the weight of pigs on the move. The MPWEADV employs the advanced ConvNeXtV2 network for robust feature extraction and integrates a cutting-edge feature fusion module. Supported by a confidence map estimator, this module effectively merges information from both RGB and depth modalities, enhancing the algorithm’s accuracy in determining pig weight. To demonstrate its efficacy, the MPWEADV achieved a root-mean-square error (RMSE) of 4.082 kg and a mean absolute percentage error (MAPE) of 2.383% in our test set. Comparative analyses with models replicating the latest research show the potential of the MPWEADV in unconstrained pig weight estimation practices. Our approach enables real-time assessment of pig conditions, offering valuable data support for grading and adjusting breeding plans, and holds broad prospects for application.

## 1. Introduction

The pork production sector holds a significant share in the global meat market, representing about 33% of worldwide meat consumption [1]. In the context of escalating population growth and shifts in dietary preferences, the efficiency and sustainability of pork production are emerging as pivotal concerns [2]. Consequently, the pork industry has been undergoing marked transformations, aiming to enhance production efficacy and meat quality while concurrently prioritizing animal welfare standards [3,4]. Within commercial swine operations, the implementation of effective management practices for swine growth and health is imperative [5,6,7]. The body weight of swine, a critical parameter for assessing both productivity and health status, is instrumental in determining the optimal market readiness of the animals. Technological advancements have led to a paradigm shift in swine-rearing practices, moving from traditional experience-based approaches to more data-driven and measurement-oriented methodologies [8]. 

The advent of precision livestock farming has brought revolutionary changes to modern animal husbandry. For instance, Cappai and colleagues enhanced the efficiency, reliability, and effectiveness of milk yield recording using RFID technology [9]. Similarly, van Erp-van der Kooij and team utilized audio and video monitoring to observe sows during farrowing, aiming to reduce piglet mortality and improve the efficiency of birth management [10]. These technologies offer novel, non-invasive, and cost-effective approaches to livestock monitoring, minimizing disruption and stress to animals by continuously collecting data [8].

In the realm of pork production systems, initial research in the application of computer vision technology focused on utilizing morphological information to estimate swine weight, as demonstrated by Schofield’s pig body imaging and processing system [11]. This approach evolved into real-time growth control systems incorporating graded visual image analysis [12]. Subsequently, dynamic weight estimation systems based on machine vision emerged, allowing for real-time, unrestricted weight estimation of pigs with an accuracy of up to 3% [13]. Jun and colleagues further enhanced the precision of weight estimation by integrating novel feature parameters with machine learning techniques [14]. However, these methods face challenges such as the inability of two-dimensional images to capture depth information, sensitivity to changes in lighting, and difficulty in accurately depicting key animal contours [15,16]. These limitations have spurred researchers to explore new technologies, such as three-dimensional imaging and advanced image processing algorithms, to overcome the constraints of existing methodologies.

Compared to 2D images, 3D images offer richer spatial information. With advancements in deep learning technology and the increased use of RGB-D sensors, research in livestock weight estimation is now focusing on depth data. For example, He created an optimized algorithm for pig weight estimation using deep learning and regression networks [16]. Nguyen employed a handheld RGB-D camera to capture RGB-D data, generate 3D point clouds, and predict pig weight using regression models such as SVR, MLP, and AdaBoost. The results indicated that SVR outperformed other models in weight prediction based on 3D characteristics [17]. Another study utilized 3D computer vision to analyze point cloud data, extract pig body measurements, and estimate weight. The research successfully developed a ridge regression equation correlating body weight, and it demonstrated that Kinect V2 is an effective tool for the livestock industry [18]. 

Although 3D cameras can make up for the lack of spatial information in 2D images, they may still face sensor noise and pixel loss issues [19]. Hence, combining RGB and depth information can enhance the feature extraction effect of the image using a multimodal deep learning approach [19,20,21]. 

In actual livestock production scenarios, numerous factors such as the livestock’s rapid movements, changes in lighting conditions, and obstruction problems affect the accuracy of the weight estimation model. Solving these problems requires enough datasets to develop more powerful and adaptive weight estimation models. However, obtaining sufficient and accurate data annotations is challenging, particularly under limited resources and the prevailing occurrence of diseases such as African swine fever [22]. 

To address these challenges, we have innovated and made improvements in three areas:

1. We have created a multimodal RGB and depth fusion architecture to capitalize on the correlation and differences between the two data types. A confidence map estimator generates feature maps and produces pixel-level reliable validity masks. These confidence maps then serve as weights for each modality of the feature map, constructing a robust weight estimation model.
2. The introduction of the FCMAE self-supervised module has significantly bolstered the backbone network’s feature extraction capabilities. Self-supervised learning mechanisms enhance the network’s ability to extract features and improve its weight estimation accuracy.
3. This study designs a pig weight estimation model that is suitable for actual production environments and corresponding scenarios. Considering the various difficulties that may be encountered in actual environments, such as the rapid movement of pigs, pig obstruction, and lighting changes, a Laplacian operator-based image blur detection algorithm, a structural similarity index measure, a deep learning-based instance segmentation network, and a keypoint detection model were introduced to address the above issue. The weight estimation model achieves promising results with an RMSE of 4.082 kg and a MAPE of 2.383% in the test set.

## 2. Materials and Methods

### 2.1. Dataset Construction

**Figure 1:**
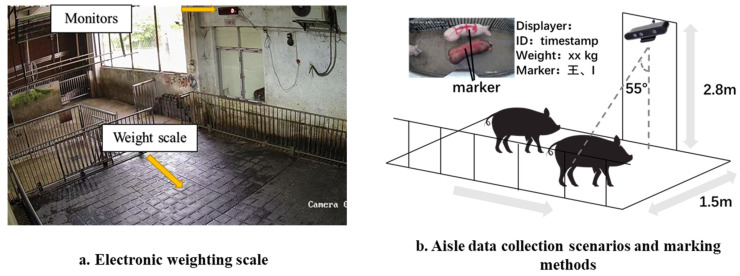
The data collection facility and scene. Panel (**a**) shows the precise ground scale used for weighing pigs, with a weight display above it. Panel (**b**) features the installation with an RGB-D camera mounted at the facility’s highest point, next to a monitor on the left. This monitor displayed an identification code for each pig, which is assigned based on a timestamp. We facilitated pig identification using a back label that corresponded to each pig’s weight on the scale.

In this study, we constructed a new dataset for analyzing the weight estimation of fattening pigs. This dataset comprises paired RGB images and corresponding depth files. For relevant data collection, our team established a data collection platform at a slaughterhouse operated by the Wenshi Group in Heyuan City, Guangdong Province. Upon arrival, workers weigh the pigs collectively and then guide them to rest in the pig pen. As illustrated in Figure 1, we installed data sampling equipment near the weighing scales. When a pig passes by, an elevated depth camera, connected to the Dell OptiPlex 3080 microcomputer, records depth, and color videos, ensuring a timely data upload to cloud computing. We utilized the Orbbec Astra Pro Plus camera model and conducted the recording using the OpenNI2 Python SDK. The RGB data resolution is 640 × 480, and the depth data resolution is 640 × 400, with a frame rate of 10 fps.

Our team conducted the data collection from June to August 2021. We randomly selected a certain number of live pigs from each batch arriving at the slaughterhouse, marked them, and weighed them five times to minimize errors due to movement. We calculated their precise weight as the average of these measurements. These pigs’ weights ranged from 74 to 154 kg, and they all belonged to the same breed. The slaughterhouse staff maintained the accuracy of the weight readings by cleaning and calibrating the scale daily. For each pig marked on its back, our team manually measured its body length, shoulder width, body height, and other physical dimensions.

**Figure 2:**
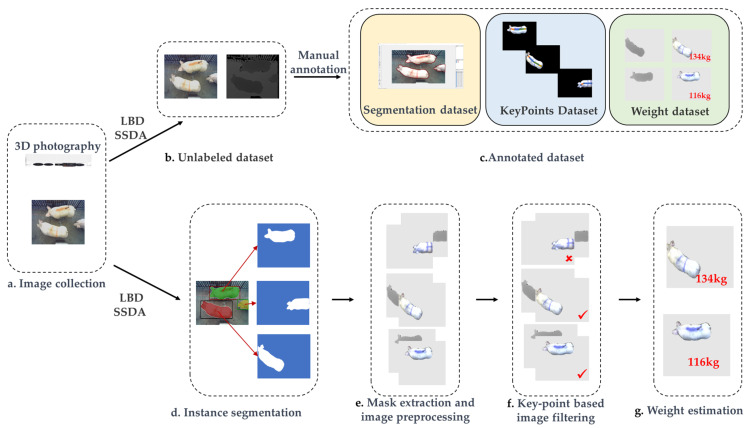
Image processing workflow. (**a**) The image acquisition step. (**b**) The original obtained RGB-D data. (**c**) The dataset after manual annotation. (**d**) The process of image segmentation. (**e**) The mask extraction process and image preprocessing. (**f**) The image selection process based on keypoints. (**g**) Weight estimation through fusion of the RGB-D data.

After collecting the original video, this paper proposes a workflow to transform the video stream data into a height-specific image set suitable for weight approximation, as illustrated in Figure 2.

**Figure 3:**
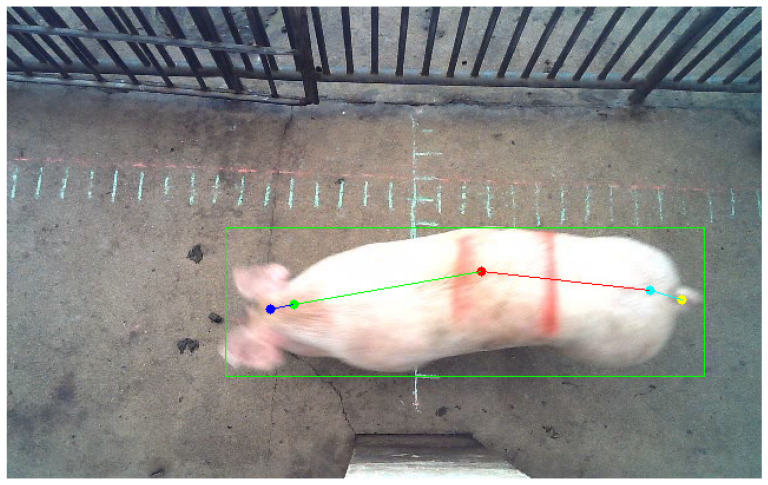
The procedure for determining the completeness of pig images through keypoint detection. If all five keypoints are detected, the image is considered included in the dataset for image weight estimation.

This paper adopts an image blur detection algorithm based on the Laplacian operator to evaluate the clarity of the image [23].

(1) $$L(x,y)=\frac{\partial^{2}f(x,y)}{\partial x^{2}}+\frac{\partial^{2}f(x,y)}{\partial y^{2}}$$

where *f*(*x*, *y*) is the intensity value of the image pixel (*x*, *y*). The Laplacian operator is overly sensitive to rapid intensity changes in the image, which often correspond to edges or boundaries. Due to the high pass nature of the Laplacian operator, it is sensitive to noise. Therefore, we use the variance of the Laplacian operator for blur detection. Considering that the value of the Laplacian operator is context-related, we scale the value of each Laplacian operator to the range of [0, 1] through minmax normalization. Finally, if the normalized Laplacian value is greater than 0.8, we define the image as a clear image. Secondly, we remove images with high similarity using a similarity detection algorithm based on the structure similarity index measure (SSIM). Finally, we employ a deep learning-based instance segmentation network to predict the pig mask instance segmentation, as referenced in [24]. Based on the number of detected keypoints mentioned in the paper, we categorize the segmented images of the pigs into two groups: complete and incomplete. To address the issue of pig bodies being obscured in the corridor scene, we have designed five keypoints: the head, neck, back, buttocks, and tail. See Figure 3 below.

**Figure 4:**
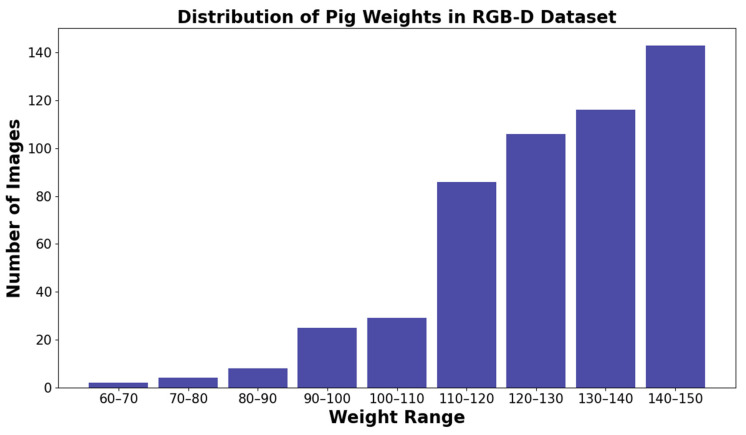
Distribution of pig weights in the RGB-D dataset.

In this study, for images identified as complete, we obtain the corresponding pig image masks and apply them to the original RGB and depth files to generate segmented images. To address noise issues and enhance depth image quality, we implement a spatial edge-preserving filter, based on Eduardo S. L. Gastal’s research [25]. Further, for cross-modal feature fusion enhancement, depth values are normalized to the 0–255 range and replicated across three channels. Both RGB and depth images are resized to 224 × 224 pixels for network efficiency. Our final dataset includes 13,594 RGB-D image pairs, with 10,875 for training and 2719 for testing, as depicted in Figure 4, which shows the weight distribution of pigs in the dataset. 

### 2.2. Methods

This study offers a pig weight estimation model, namely MPWEADV, which utilizes the RGB-D fusion framework to estimate pig weight in alley environments. The model incorporates segmented RGB images and corresponding depth images of a single pig and couples them with the validity mask, which the confidence map predictor generates. This combination results in an RGB-D feature fusion at various scales. Moreover, this paper includes the design of a downstream weight estimation task network, containing an FPN neck and a pig head regression task module, which is responsible for completing the pig weight estimation. To confirm whether the model effectively learns the necessary features for weight estimation, this paper adopts a CAM module for appropriate verification. 

#### 2.2.1. Feature Extraction Network Master Module ConvNeXtV2

Self-supervised learning (SSL) is a machine learning training method that utilizes vast amounts of unlabeled data to enhance model performance in terms of prediction through internal feature learning. One of the recent SSL strategies is the utilization of masked image models, like masked autoencoders, which have had a considerable impact on visual recognition as a neural network pretraining framework [26]. Nevertheless, the asymmetric encoder–decoder design of masked autoencoders prevents their direct application to convolutional neural networks. To address this issue, researchers have proposed the ConvNeXtV2 network that features a fully convolutional masked autoencoder (FCMAE) module for self-supervised pretraining, which significantly improves the visual feature learning ability [27]. 

The FCMAE module operates in a fully convolutional manner and randomly removes 60% of the 32 × 32 blocks from the input image to process the visual effect with a high mask rate random masking strategy. As an effective solution to prevent the model from learning the masked area information, the FCMAE module applies the ConvNeXt encoder and treats the masked image as a two-dimensional sparse pixel array.

Moreover, sparse convolution operates only on visible data points, preserving the two-dimensional image structure. The decoder is a lightweight ConvNeXt block that reconstructs the image based on encoded pixels and mask labels. The reconstruction target relies on the mean-square error (MSE) between the reconstructed and target image, applying the loss only to the masked blocks.

**Figure 5:**
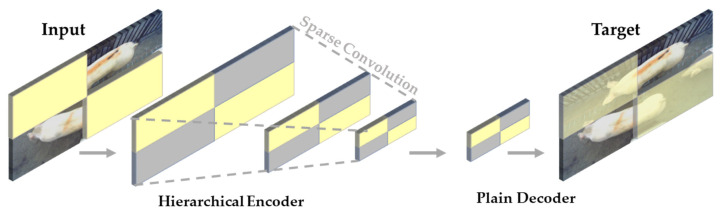
A schematic diagram of the fully convolutional masked autoencoder (FCMAE) architecture. The FCMAE employs a ConvNeXt encoder based on sparse convolution and a lightweight ConvNeXt block decoder. The diagram splits the image into two parts: the encoder on the left and the decoder on the right. The encoder takes the original image and its corresponding mask as inputs, producing an encoded feature map and a mask token as outputs. Conversely, the decoder uses the encoded feature map and mask token to reconstruct the image. Arrows between the encoder and decoder illustrate the information flow direction, indicating that the loss function is computed solely in the mask region.

The FCMAE module boasts an asymmetric encoder–decoder structure where the encoder processes only visible pixels to avoid masked area information leakage. Overall, the FCMAE module promotes effective masked image modeling through masking, sparse convolution, and MSE based reconstruction. See Figure 5 below.

The ConvNeXtV2 network introduces the global response normalization (GRN) layer, which effectively improves feature competition and resolves the feature collapse problem within the ConvNeXt network. The GRN layer utilizes a cosine similarity-based analytical approach to enhance network performance. After pretraining, the ConvNeXtV2 network undergoes supervised finetuning and attains outstanding outcomes. Extensive experimental results demonstrate that the ConvNeXtV2 network significantly enhances ConvNeXt performance across diverse downstream tasks [27]. 

#### 2.2.2. Weight Estimation Backbone

Visual methods for estimating livestock weight commonly employ RGB-D images, point clouds, and RGB images to achieve precision, robustness, and real-time performance. Unfortunately, most research only focuses on either RGB or depth image data, which poses difficulties in obtaining strong feature representations in real-life scenarios and often results in limited recognition accuracy. Improved automatic recognition accuracy is particularly crucial in modern intelligent breeding. While multimodal neural networks can enhance accuracy and robustness by leveraging the complementary nature of color and depth information, there is a shortage of research on feature fusion at varying stages and scales. 

**Figure 6:**
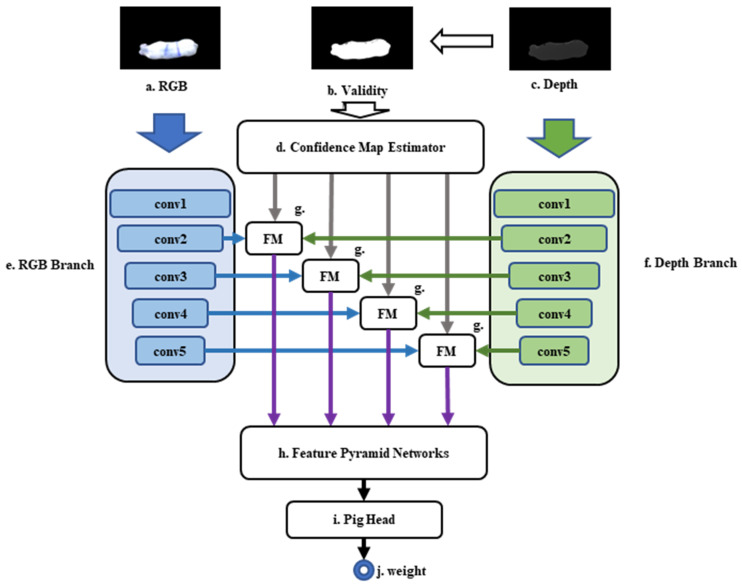
The RGB-D fusion backbone network. (**a**) The RGB image stream; (**b**) The confidence map image stream based on depth information extraction; (**c**) the depth information image stream; (**d**) the confidence map estimator module; (**e**) the main feature extraction module of the RGB image information; (**f**) the main feature extraction module of the depth image information; (**g**) The feature fusion module; (**h**) the feature pyramid feature fusion module; (**i**) the weight estimation linear head module; and (**j**) the weight value of the obtained pig.

Hence, this study advocates a multimodal feature fusion backbone extraction network that hinges on V2ConvNeXtV2 to enhance the resilience of feature learning through the fusion of RGB-D features. This approach captures the complementary characteristics of RGB and depth images, thereby achieving more precise pig weight estimation. The network’s fundamental principle is a hierarchical fusion of RGB and depth information with the added emphasis on reweighting depth features. Figure 6 illustrates the RGB-D skeleton network’s functionality. 

In this study, hierarchical RGB-D information fusion consists of three input branches and fusion modules. 

RGB branch. As depicted on the left side of Figure 6, color information enters through Figure 6a and undergoes processing by the main feature extraction module shown in Figure 6f. This module generates feature maps of different scales, which the feature fusion module then fuses, as seen in Figure 6h.

Depth branch. As illustrated on the right side of Figure 6, depth information enters through Figure 6c and passes through the main feature extraction module depicted in Figure 6g. This module creates feature maps of various scales, which are subsequently combined in the feature fusion module displayed in Figure 6h.

Confidence branch. The sequence for this branch, shown in the center of Figure 6, starts by forming a complete matrix with the same dimensions as the depth information image, as presented in Figure 6c. Positions in the depth chart with a value of 0.0, indicating missing or questionable depth values, are replaced with 0 in the validity mask corresponding to the location on the active mask. This process generates a single-channel validity mask feature map that feeds into the confidence predictor, as shown in Figure 6e. The confidence map estimator (CME) then uses depth data to accurately disperse the validity mask feature map at the pixel level through five convolutions, producing an output feature map for the feature fusion module in Figure 6h.

Fusion module. The design of the fused module in this study considers the input data flow from the three branches. Initially, we multiply the validity mask feature map (created based on Figure 6e) with the depth feature map of the corresponding scale (acquired from Figure 6g) to enhance the feature map of the high-confidence region. Then, we concatenate the color and depth feature maps and obtain the fused feature map through a 1 × 1 convolution. This design fully exploits the information provided by the color and depth data to maximize the model’s performance. The fusion formula depicted in the pseudocode (see Algorithm 1) accomplishes feature fusion and dimension reduction through pixel by pixel addition and subsequent convolutional layers.
**Algorithm 1:** Pseudo-code of Rgb/Depth Feature Fusion**Inputs:**   RGB frame F*_rgb_*.   Depth frame F*_depth_*.   Confidence mask *val_mask*   Confidence map predictor *CME*.   Convolution layers conv1, conv2, conv3, conv4.   Feature map *FM*.   Feature fusion layer *FL***Output:** Feature map after 2D/3D feature fusion *FLs_out**FM_rgb_* ← *conv1_rgb*(*F_rgb_*);2.*FM_depth_* ← *conv1_depth*(*F_depth_*);3.Mask_validity ← *CME*(*val_mask*);4.*CMs* ← *CM*(*val_mask*) #Divide the predictor based on hierarchical size5.**for** *i* = 2 **to** 4 **do**6. *FM*[*i*]*_depth_* ← *conv*[*i*]*_depth_*(*FM_depth_*) × *CM*[*i*];7. *FM*[*i*]*_out_* ← *concat*(*FM*[*i*]*_depth_*, *FM_rgb_*);8.**for** *i* = 2 **to** 4 **do**9. *FL*[*i*]*_out_* ← *FL*[*i*](*FM*[*i*]*_out_*);

#### 2.2.3. Estimated Head and Neck Network Design

This study proposes a design strategy, grounded in the feature pyramid network (FPN) [28], that aims to improve the accuracy of weight estimation tasks. This enhancement is achieved by utilizing feature maps across various scales. 

The task of weight estimation is bifurcated into two primary processes: feature extraction and downstream tasks. The network’s backbone is tasked with extracting features from image-based data, while the head is responsible for weight estimation, with a particular focus on minimizing overfitting. In the design specific to this study, the backbone initially extracts feature maps across four different scales to serve as inputs. 

Subsequently, we conduct a feature fusion process using the FPN module, which plays a crucial role within the network as a ‘connector’ between feature maps of different scales. To mitigate overfitting, we select the P2 feature map, with dimensions of 160 × 160 × 256, from the FPN module’s output for feature channel data extraction. After extraction, we feed this data into the weight estimation head. 

**Figure 7:**
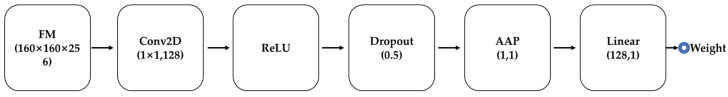
Weight estimation head network architecture.

The input feature map Fm is processed by a 1 × 1 convolution layer (Conv1 × 1(Fm)), followed by ReLU activation and dropout layers for feature extraction and dimension reduction. Finally, the weight estimation outputs are generated using an adaptive average pooling layer (AAP) and a fully connected layer, as depicted in Figure 7. 

To rigorously evaluate the performance of the regression model, we have adopted a set of key indicators to measure the accuracy and predictive power of the model. 

(1) Mean absolute error (MAE). MAE measures the average degree of error in a set of predictions regardless of their direction, and it is a linear fraction, leading to equal weighting of individual differences.

(2) $$\mathrm{MAE}=\frac{1}{B_{n}}\sum_{i=1}^{B_{n}}|y_{i}-y_{i}^{\prime }|$$

where *y_i_* is the true weight, *y’_i_* is the predicted weight, and *B_n_* represents the batch size. 

(2) Mean absolute percentage error (MAPE). MAPE computes the mean absolute error (MAE) as a percentage, providing an intuitive understanding of the error expressed in percentage form.

(3) $$\mathrm{MAPE}=\frac{1}{B_{n}}\sum_{i=1}^{B_{n}}\left|\frac{y_{i}-y_{i}^{\prime }}{y_{i}}\right|$$

where *y_i_* is the true weight, *y’_i_* is the predicted weight, and *B_n_* represents the batch size. 

(3) Root-mean-square error (RMSE). RMSE is a popular measure that gauges the difference between values predicted by the model and observed values. RMSE is a commonly used measure to measure the difference between the values predicted by the model and the observed values. Before taking the average of the squared differences, it squares the difference which, in turn, assigns higher weights to larger differences.

(4) $$\mathrm{RMSE}=\sqrt{\frac{1}{B_{n}}\sum_{i=1}^{Bn}{\left(y_{i}-y_{i}^{\prime }\right)}^{2}}$$

where *y_i_* is the true weight, *y’_i_* is the predicted weight, and *B_n_* represents the batch size. 

(4) Coefficient of determination (R^2^). We use the coefficient of determination to measure how well the model captures the variability in the data. A value close to one indicates a strong fit. It is computed as

(5) $$R^{2}=1-\frac{\sum_{i}{\left(y_{i}-y_{i}^{\prime }\right)}^{2}}{\sum_{i}{\left(\bar{y_{l}}-y_{i}\right)}^{2}}$$

where *y_i_* is the true weight and *y’_i_* is the predicted weight. 

### 2.3. Training Setup

**Figure 8:**
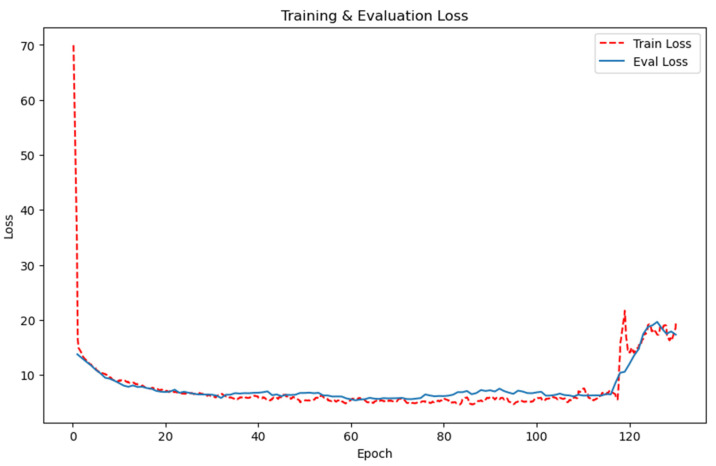
Training and testing loss curves of the model.

For this experiment, we employed the Determined AI framework, conducting our research under Python version 3.8.10 and PyTorch version 1.12 environments [19,20]. We used OpenCV for image filtering and visualization. Our server infrastructure boasted an Intel Xeon^®^ Silver 4214R 2.4 GHz processor with 128 GB memory and a 24 GB NVIDIA Ampere A30 Graphics Processor. We carried out the training process over 130 epochs in a computational environment equipped with CUDA 11.3.1, running on Ubuntu 20.04 LTS. We chose AdamW as the optimizer, set the learning rate to 1.25 × 10−4, the weight decay to 0.1, and the batch size to 20. Figure 8 displays the training and testing loss curves of the model. The distinct paths traced by these curves demonstrate the model’s convergence and its robustness under various scenarios. Notably, we deemed the model weights recorded during the 110th epoch as optimal for the context of this article.

## 3. Results and Discussion

### 3.1. Comparing Single Modal Networks

To verify the effects of other models in the same scenario, this study references earlier studies, reproduces a regression model built on pig body size parameters obtained from depth images for single modality situations [18], and uses another method that is a deep learning model directly built on depth value images [16]. Using the already measured data, we obtained body length (*l*), shoulder width (*w*), and body height (*h*) information, with the measurement unit in centimeters. From this physical dimension information, we calculated both a linear regression model and a second-degree polynomial regression model, yielding the following formulas:

For the linear model, the regression equation is the following:

(6) weight=1.3127×l+1.3267×w+0.0814×h−102.4994

The regression equation for a quadratic polynomial model is the following:

(7) $$\begin{array}{ll} \mathrm{weight}= & -0.0098l^{2}-0.1351l\cdot w-0.0917l\cdot h+9.1156l\\ & +0.0056w^{2}-0.0075w\cdot h+16.1634w-0.0841h^{2}\\ & +15.3916h-1369.4085 \end{array}$$

Moreover, this study replicated a more sophisticated single modality weight estimation method. This alternative method incorporates ResNet and BotNet blocks, deploying a dual branch and parallel fully connected layer blocks strategy following preprocessing based on depth images [16]. We undertook the replication of the study for the purposes of validation and to build upon the foundational work established by the original authors. Although the authors chose not to release their source code publicly, a valuable effort by the open-source community on GitHub provided us with a suitable starting point [29]. Our replication efforts, detailed in Table 1, aimed not only to verify the original findings but also to explore the comparative effectiveness of single modality methods utilizing depth values versus those based on body measurements.

The results could be due to inconsistencies in pig postures, which introduce complexities to the weight estimation process. This section provides a comparative analysis of the linear regression, quadratic regression, and BotNet + DBRB + PFC methods, focusing on key indices such as the mean absolute error (MAE), mean absolute percentage error (MAPE), root-mean-square error (RMSE), and R-squared (R^2^).

Our comparison reveals that the quadratic regression is adept at capturing the fundamental trends of the data, as evidenced by its performance indicators. While linear regression and BotNet + DBRB + PFC methods each offer distinct benefits, their specific limitations reduce their effectiveness for this particular dataset. Moreover, our proposed approach integrates RGB and depth data, yielding metrics such as an MAE of 2.856, MAPE of 2.383%, RMSE of 4.082, and an R^2^ of 0.901. This significant improvement in prediction accuracy highlights the strengths of multimodal learning and the importance of using varied modalities for added insight. The subtle differences in performance shed light on the potential upsides of applying multimodal learning strategies, especially when dealing with diverse data types.

### 3.2. Comparing Multimodal Networks

In the review of the published literature, various multimodal papers relevant to live pig weight estimation have been identified [30]. This study references these works, undertaking replication attempts.

Given that the original authors have not released the source code, we based our replication in this study on Microsoft’s open-source code available on GitHub [31]. Furthermore, we incorporated the network fusion methods proposed by the authors [30]. Drawing from these references, we define the elements of our architecture as follows:

Early fusion (Early). This strategy combines data at the input layer, forming four-channel inputs (RGB-D) from both RGB and depth images.

Late fusion (Late). Implemented at higher network levels, this strategy processes each input mode independently and then merges the results. Notably, the late fusion model proposed here lacks a confidence map estimator despite having separate RGB and depth branches.

Confidence map estimator fusion (Est). As an advanced approach, this method employs convolutional neural networks to generate pixel-level validity maps, assigning reliability scores to depth information feature maps. We then use these maps to weight individual feature mappings.

We present comparative findings in Table 1. These findings underscore that the proposed method delivers superior performance metrics—mean absolute error (MAE), mean absolute percentage error (MAPE), root-mean-square error (RMSE), and R-squared (R^2^)—within the context of this study. However, these results do not unequivocally establish the proposed method’s overall superiority; they simply indicate its relative effectiveness in addressing the unique challenges of the current scenario. Moreover, when benchmarked against the Swin-T + CAB model, the proposed method shows improvements across metrics, including significant reductions in error metrics (MAE, MAPE, and RMSE) and an increase in the determination coefficient (R^2^), indicating heightened precision and model fitting under certain conditions. Although Swin-T + CAB has its advantages, the proposed method adeptly handles the specifics of this scenario, leading to enhanced performance.

At present, the proposed method potentially offers superior performance exclusively within the defined study scenario. This underscores the need for enhanced emphasis on contrasting advantages across diverse models under different situational contexts, which is a strategy that could foster algorithmic advancements. It is vital to parse the results meticulously, recognizing model-specific contextual constraints and thereby bolstering the proposed method’s generalizability.

### 3.3. Discussion

#### 3.3.1. Results Analysis

To facilitate a broader comparison, this study draws inspiration from Pezzuolo [32] to design linear and quadratic regression models. The Table 1 results reveal superior performance by the quadratic multivariate regression model.

However, the dataset constructed for this research did not yield optimal outcomes. The generated errors stem from measurement inaccuracies, vague measurement standards, and challenges navigating optimal pig postures due to the absence of constraints on pig movement. These unusual postures are the predominant factor compromising the weight prediction performance [33].

The evolution of deep learning’s foundational capabilities has catalyzed progress in noncontact weight measurement. For instance, Jun integrated novel feature parameters with machine learning to enhance weight estimation [14].

However, the conversion of 3D world camera mapping to a 2D space may result in the loss of potentially crucial height information required for weight estimation tasks. Consequently, several researchers propose the incorporation of depth image information [16]. Still, a lone depth image may lack other potentially crucial textures, colors, and datasets for weight estimation tasks. This deficiency in detailed target object information inflates the likelihood of misidentifying objects of great similarity [34]. Most earlier measurements primarily focused on a single mode, thereby neglecting other modes’ supplementary information. Employing multimodal representation across RGB and depth modes can efficiently leverage multimodal information to deliver more precise pig weight estimation [30].

The comparative experiment in Table 1 suggests that, versus a single mode and presently published technologies, our proposed method significantly decreases MAE, MAPE, and RMSE in the dataset for this research’s constructed scenes, partially affirming our proposed method’s efficacy. Notably, the data do not feature any manually selected or posture constraints—a fact that evidences our technique’s effectiveness in estimating pig weight despite absent posture constraints.

**Figure 9:**
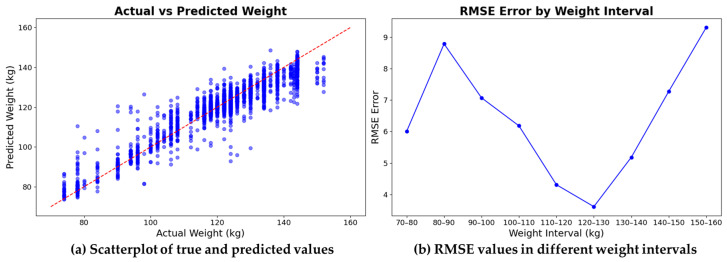
Weight estimates and true weight values of the proposed method, and RMSE error plots at different intervals.

Figure 9a presents the actual and predicted weights, displaying a significant overall correlation. Albeit some predictions deviate from actual values due to posture, multiple pigs in proximity, and pigs at the shooting range edge, resulting in posterior visibility issues. Figure 9b exhibits the RMSE value’s relationship with pig weight. Within a certain range, RMSE increases as weight increases—a likely outcome of the scarce data for particularly light (below 110 kg) or heavy (above 140 kg) pigs in the loose regional dataset, causing network prediction performance fluctuations.

**Figure 10:**
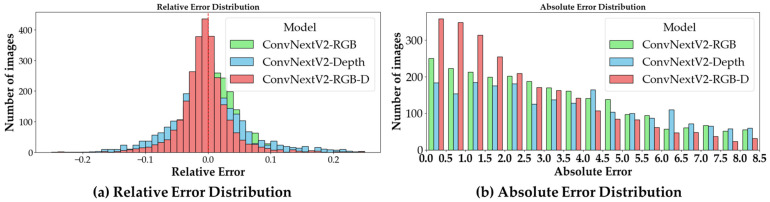
Relative error distribution and absolute error distribution.

The Figure 10 shows the distribution of relative error and absolute error from our network. Compared to ConvNextV2 based on the single RGB modality and ConvNextV2 based on the single depth modality, our proposed method presents a more concentrated distribution in the lower value range. This suggests that our method has stronger robustness compared to the baseline methods, which demonstrates the complementary properties of information from different modalities.

#### 3.3.2. Impact of Swine Postural Dynamics on Weight Estimation

While other models demonstrate robust performance, they failed to produce accurate weight estimation results when applied to the dataset presented in this study. The lack of consistent posture among the pigs in this dataset likely contributes significantly to inaccurate weight predictions [14]. This highlights the need to investigate the influence of posture on weight estimation accuracy. To quantify the posture distribution within the dataset used in this study, we introduce the concept of a ‘posture score’. This score is calculated based on five anatomical landmarks of the subject: the head (a), the neck (b), back (c), buttocks (d), and tail (e). To establish a positive correlation between the score and image quality, we compute the cosine values of three pairs of line segments ((l_ab_, l_bc_), (l_bc_, l_cd_), and (l_cd_, l_de_)) on a two-dimensional plane and convert them into acute angle values. The calculation formula is as follows:

(8) $$\theta =\cos^{-1}\left(\frac{\vec{u}\cdot \vec{v}}{\left|\left|\vec{u}\right|\right|\times \left|\left|\vec{v}\right|\right|}\right)$$

To maintain the acuteness of θ, we further refine it as follows:

(9) $$\theta =\min(\theta ,180^{{}^{\circ}}-\theta )$$

Then, we use these acute angle measurements to calculate the posture score. For degrees containing n angles, the calculation formula for the score is as follows:

(10) $$\mathrm{Score}=\sum_{i=1}^{n}\left(\frac{100}{n}\times \left(1-\frac{\theta_{i}}{90}\right)\right)$$

**Figure 11:**
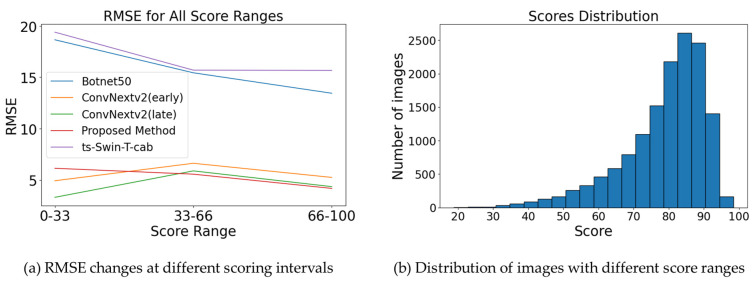
RMSE plots of different models under image score intervals, and the distribution of the number of images under image score intervals.

Figure 11a displays the root-mean-square error (RMSE) metrics corresponding to several posture score points, confirmed with diverse models. The figure indicates a positive correlation between posture and estimation accuracy, demonstrating that as the posture score escalates, the model error tends to diminish.

In the context of posture estimation, with scores between 66 and 100, our method aligns closely with the performance of other integrated approaches. This suggests that, in ideal conditions, the ConvNeXtV2 network excels in extracting key features reliably. In the moderate score range (33–66), our approach continues to outperform similar methods in efficiency. However, when posture scores fall below 33, all models, including ours, show notable inconsistencies in RMSE measurements. This may be linked to the limited examples of low-score images in our dataset, as illustrated in Figure 11b, potentially impacting model stability. Moreover, a review of Figure 11b highlights the positive impact of our image preprocessing steps, enhancing the overall dataset quality.

Our current focus is on segments of the pig weight estimation task with high posture estimation scores, particularly those scoring sixty-six or above. A deeper exploration of the correlation between posture and weight prediction, using quantitatively defined methodologies, could significantly advance pig weight estimation technology.

Therefore, our proposed method aligns better with the estimation accuracy demands inherent to the scenario, especially for postures with a score of 66 or higher.

#### 3.3.3. Impact of Backbone

The domain of deep learning displays a range of backbone networks, each offering distinctive feature extraction and classification capabilities. For instance, ResNet utilizes residual connections to deepen the network and augment its performance, while ResNeXt leverages grouped convolution to enhance network efficiency and scalability [35]. ConvNeXt bolsters the model’s expressiveness and interpretability via multichannel convolution [36]. Building upon ConvNeXt, ConvNeXtV2 further optimizes the model’s performance and speed [27].

To assess the impact of these networks on weight estimation performance, this study conducted a comparative analysis across several deep learning-based backbone networks. According to Table 2, ResNet50 (Est) and ResNeXt50 (Est) demonstrate comparable performance; however, ResNeXt50 (Est) exhibits a slight advantage in the RMSE metric, suggesting its marginal superiority in weight estimation due to its refined architecture. On the other hand, BotNet + DBRB (Est), incorporating the feature fusion architecture proposed in this study, shows a notable improvement compared to its original structure. While the ConvNeXt(Est) network possesses high computational complexity, its performance metrics MAE and MAPE fall short of expectations, indicating that complex network architecture does not guarantee optimal performance. In comparison to alternative approaches, our proposed method achieves exceptional performance across MAE, MAPE, RMSE, and R^2^ measures. Specifically, our method achieved an MAE of 2.856 kg, a MAPE of 2.383%, reduced RMSE to 4.082, and attained an impressive R^2^ value of 0.901, demonstrating its significant advantage in terms of weight estimation accuracy and reliability.

#### 3.3.4. Impact of the Fusion Method

To evaluate the influence of different modalities and fusion strategies on weight estimation accuracy, we performed a comparative analysis using various configurations: ConNeXtV2 (null) with RGB, ConNeXtV2 (null) with Depth, ConNeXtV2 (Early) with RGB + Depth, ConNeXtV2 (Late) with RGB + Depth, and our proposed ConNeXtV2 (Est) method with RGB + Depth. The experimental results are summarized in Table 3. The ConNeXtV2 (null) model exhibits varying performance across modalities: the RGB modality achieved an MAE of 4.355 and an R^2^ of 0.762, while the depth modality demonstrated an MAE of 6.163 and a significantly lower R^2^ of 0.538. This suggests that the RGB modality provides more accurate weight estimations in this model configuration. Both ConNeXtV2 configurations that incorporate RGB and depth modalities showed improved performance. Moreover, our proposed method utilizing these modalities achieved the most favorable results across all metrics.

#### 3.3.5. Limitations

While our work presents significant advancements, it also encompasses certain limitations. Firstly, we have not comprehensively evaluated the impact of varying shooting locations and angles on pig weight estimations, nor have we conducted an in-depth comparative analysis. Future endeavors will focus on addressing this research gap. Secondly, our dataset excludes images with severe occlusion where keypoints are obscured. Moving forward, we aim to explore more effective strategies to improve weight estimation accuracy for such excluded pigs, rather than simply eliminating them. The third limitation concerns our equipment selection. In contrast to the widespread use of Microsoft Kinect DK (Redmond, Washington, DC, USA) and Intel RealSense cameras (Santa Clara, CA, USA), we employed the Orbbec Astra Pro Plus camera (Shenzhen, China). However, we have not yet investigated the potential influence of shooting accuracy variations unique to these devices on weight estimation accuracy. Finally, our dataset incorporates corridor scenes. Although we have successfully implemented unconstrained weight estimation for free-roaming pigs, the dataset solely comprises data from slaughterhouses, neglecting a broader weight range. To enhance the dataset’s representativeness, we envision future improvements that encompass collecting comprehensive data across various weights, thereby augmenting the model’s generalizability.

## 4. Conclusions

In this study, we developed a novel RGB-D feature fusion module specifically tailored to address the challenges of pig weight estimation. We introduced the ConvNextV2 network, incorporating a self-supervised module, FCMAE, which augments the feature extraction capabilities of the backbone network through self-supervised learning. Both RGB and depth data are fused at the regression layer for accurate weight estimation. We constructed a dedicated RGB-D data collection platform specifically designed for pig weight estimation experiments. To validate the efficacy of our proposed method, we meticulously replicated several advanced, recently published single-stream and double-stream feature fusion weight estimation networks for comprehensive comparative analysis. Additionally, this study introduces a novel scoring system to assess pig image quality, effectively quantifying the impact of pig posture on weight estimation results. Our findings demonstrated that our proposed method outperformed all evaluated methods, achieving superior performance in this dataset, with a remarkable root-mean-square error (RMSE) of 4.082 kg. Future research endeavors include enhancing the dataset to facilitate more comprehensive design and implementation of pig weight estimation models. Our research is steadfastly directed toward meeting large-scale commercial application demands and promoting welfare-oriented farming practices.

## Figures and Tables

**Table 1:** Comparison results of the RGB-D image dataset.

Method	Modality	Parameters	FLOPS	MAE	MAPE	RMSE	R^2^
Linear Regression	Depth	-	-	8.014	7.235%	10.151	0.733
Second Degree Regression	Depth	-	-	7.350	6.530%	8.380	0.818
BotNet + DBRB + PFC	Depth	29.83 M	23.24 G	8.169	7.336%	10.606	0.457
Ts-Swin-T + CAB	RGB + Depth	53.59 M	8.77 G	10.877	10.145%	15.508	−0.032
Proposed Method	RGB + Depth	202.97 M	38.55 G	2.856	2.383%	4.082	0.901

**Table 2:** The results of various backbone architectures for the dual-stream fusion network..

Method	Modality	MAE	MAPE	RMSE	R^2^
ResNet50 (Est)	Rgb + Depth	4.490	3.848%	5.846	0.684
BotNet + DBRB (Est)	Rgb + Depth	4.925	4.244%	6.924	0.566
ResNeXt50 (Est)	Rgb + Depth	4.150	3.443%	5.588	0.742
ConvNeXt (Est)	Rgb + Depth	5.573	4.934%	7.288	0.595
Proposed Method	Rgb + Depth	2.856	2.383%	4.082	0.901

**Table 3:** The results of various fusion methods for the Dual-Stream Fusion Network.

Method	Modality	MAE	MAPE	RMSE	R^2^
ConNeXtV2 (null)	Rgb	4.355	3.734%	5.695	0.762
ConNeXtV2 (null)	Depth	6.163	6.540%	8.338	0.538
ConNeXtV2 (Early)	Rgb + Depth	3.996	3.404%	5.480	0.837
ConNeXtV2 (Late)	Rgb + Depth	4.016	3.387%	5.469	0.832
Proposed Method	Rgb + Depth	2.856	2.383%	4.082	0.901

## Data Availability

Data are contained within the article.

## Abbreviation

MPWEADV	Moving pig weight estimate algorithm based on deep vision
Early	Early fusion
Late	Late fusion
Est	Confidence map estimator fusion
FLOPs	Floating point operations
FPS	Frames per second
LBD	Laplacian-based detection algorithm for detecting image blur
SSDA	Structural similarity detection algorithm based on the structural similarity index measurement
Param	Parameters
FM	Fusion method
BotNet + DBRB + PFC	Botnet block followed by a parallel fully connected layer block
Ts-Swin-T + CAB	Two-stream Swin transformer + CAB
